# The increased functional connectivity between the locus coeruleus and supramarginal gyrus in insomnia disorder with acupuncture modulation

**DOI:** 10.3389/fnins.2023.1131916

**Published:** 2023-04-20

**Authors:** Zhaoyi Chen, Tongfei Jiang, Xuejiao Yin, Bin Li, Zhongjian Tan, Jing Guo

**Affiliations:** ^1^Beijing Key Laboratory of Acupuncture Neuromodulation, Department of Acupuncture and Moxibustion, Beijing Hospital of Traditional Chinese Medicine, Capital Medical University, Beijing, China; ^2^Department of Radiology, Dong Zhimen Hospital, Beijing University of Chinese Medicine, Beijing, China

**Keywords:** insomnia disorder, acupuncture, locus coeruleus, resting-state functional connectivity, fMRI, neuroimaging

## Abstract

**Background:**

Insomnia disorder (ID) seriously affects the quality of people’s daily life, and acupuncture is an effective therapy for it. As an essential component of the upward activation system, the locus coeruleus (LC) plays a crucial role in sleep–wake regulation, its aberrant functional connectivity (FC) is found to be involved in ID. The purpose of this study was to explore the modulation effect of acupuncture on the resting state FC of LC in ID patients.

**Methods:**

60 ID patients were recruited and randomly assigned to real acupuncture (RA) or sham acupuncture (SA) treatment. Resting-state functional magnetic resonance imaging (fMRI) data were collected before and after the treatment. With LC as the region of interest, the FC method was adopted to examine acupuncture-related modulation of intrinsic connectivity in ID patients. The Pittsburgh Sleep Quality Index (PSQI), Hyperarousal Scale (HAS), and actigraphy were used to assess sleep quality and cortical hyperarousal states. Associations between clinical outcomes and FC features were calculated using Pearson’s correlation analysis.

**Results:**

The improvement in sleep quality and hyperarousal in the RA group was greater than that in the SA group. After treatment, the FC between the LC and left inferior frontal gyrus (IFG) decreased in the RA group. The FC between the LC and left insula and supramarginal gyrus (SMG) was higher in the RA group. The change of LC FC values with the SMG was negatively associated with the change in PSQI scores.

**Conclusion:**

Acupuncture can modulate FC between the LC and IFG, insular gyrus, and SMG. This may imply the potential mechanism of acupuncture treatment for insomnia.

## 1. Introduction

Insomnia disorder (ID) is the inability to initiate and maintain sleep despite adequate opportunity for sleep, accompanied by significant daytime dysfunction (Battle, 2013). With a prevalence rate of 30–35% and an annual incidence rate of 7–15% (Morin et al., 2015), ID affects our mental and physical health widely (Pigeon et al., 2017; Khan and Aouad, 2022).

As a kind of complementary therapy, acupuncture is widely recognized as a potentially effective treatment method for ID. It has shown a positive effect on improving sleep quality (Zhang et al., 2020; Zhao et al., 2021) and alleviating the symptoms of anxiety and depression brought on by insufficient sleep (Liu C. et al., 2021). While the potential mechanisms remain to be elucidated.

The hyperarousal theory, which proposes increased activation in specific brain regions in insomniacs, is largely recognized as an explanation for the origin of insomnia (Bonnet and Arand, 1997). The locus coeruleus (LC), a crucial part of the ascending activation system, is a major norepinephrine (NE) producer and projects a large number of neurons to the spinal cord and cortex (Benarroch, 2018). LC-NE neurons remain active while awake, and at a resting state during sleep, especially during the rapid eye movement phase (Eschenko and Sara, 2008; Eschenko et al., 2012). Therefore, LC was regarded as an important contributor to promoting wakefulness and maintaining sleep.

Studies using fMRI have shown that LC plays an important role in insomnia. The functional connectivity (FC) between the LC and the posterior cingulate, thalamus, and caudate nucleus alters with the awakening state (Song et al., 2017). In insomniacs, the FC of LC increased in areas of the sensory cortex and default mode network (DMN) while decreased in the prefrontal cortex (Gong et al., 2021). Acupuncture has been shown to regulate the electrophysiology of LC in rat models of insomnia (Lee and Kim, 2017). We hypothesize that regulating the functional activity of LC may be one of the mechanisms for acupuncture to improve sleep. This study aims to investigate the modulation effect of acupuncture on the FC of the LC in ID.

## 2. Methods

### 2.1. Study design and ethical approval

This is a single-center, randomized, sham-controlled trial. The trial was registered on the Chinese Clinical Trial Registry (ChiCTR1800015282, http://www.chictr.org.cn/index.aspx) and was approved by the Medical Ethical Committee of the Beijing Hospital of Traditional Chinese Medicine (2018BL-002-02). Prior to the beginning of the trial, each participant was required to sign an informed consent form.

### 2.2. Participants

Sixty right-handed subjects who scored a total of 8 or more in the Pittsburgh Sleep Quality Index (PSQI) and had a diagnosis of insomnia disorder according to the Diagnostic and Statistical Manual of Mental Disorders (5th edition) were recruited for the trial. Those with an unstable medical condition, a diagnosis of anxiety, depression, or other sleep disorders, such as sleep apnea and restless leg syndrome, a contraindication for MRI, an addiction to drugs or alcohol, or a history of acupuncture therapy within 1 month were excluded. All eligible participants were randomly assigned to either the real acupuncture (RA) or sham acupuncture (SA) group.

Out of 60 subjects recruited for the study, ten patients dropped out due to COVID-19 or scheduling conflicts. Fifty patients (26 in the RA group and 24 in the SA group) who completed the two fMRI scans were included for data analysis.

### 2.3. Interventions

Patients in the RA group received acupuncture at Baihui (GV-20), Shenting (GV-24), Benshen (GB13), Sishencong (EX-HN1), bilateral Sanyinjiao (SP-6), bilateral Neiguan (PC-6), and bilateral Shenmen (HT-7). The selection of these acupuncture points was based on our previous clinical study, which found that these points improved sleep quality and daytime function in patients with insomnia (Guo et al., 2013). Needles were entered 10 mm horizontally for GV-20, GV-24, GB13, and EX-HN1, 5 mm vertically for HT7 and PC6, and 10 mm vertically for SP6, all while twisting the needle to induce a Deqi sensation, a sensation that includes numbness, soreness, and distension.

In the SA group, acupoints not related to insomnia treatment were selected, such as Binao (LI-14), Shousanli (LI-10), Yangchi (TE-4), Waiguan (TE-5), Fengshi (GB-31), Liangqiu (ST-34), and Futu (ST-32) (all bilateral). These points are often used to treat local tissue ailments and have little therapeutic effect on insomnia according to literature search. Needles were inserted superficially without manual stimulation.

All subjects received three 30-min acupuncture sessions per week for 4 weeks.

**Figure 1:**
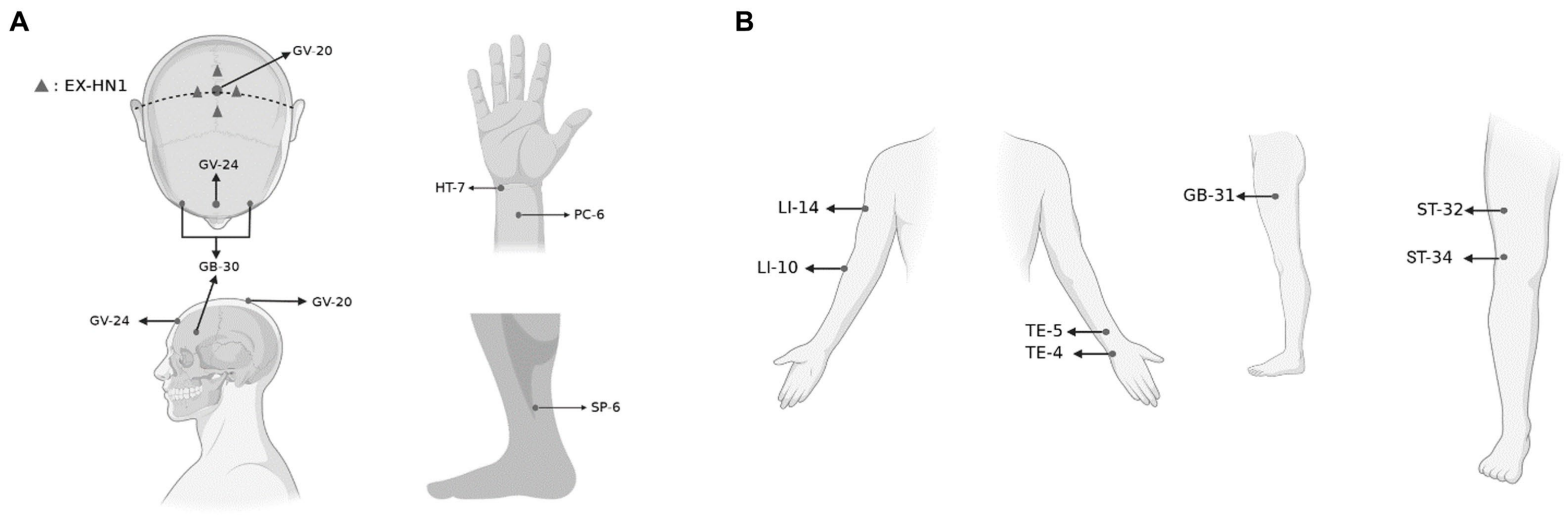
The acupoints selected in the trial. **(A)** The acupoints selected in the RA group and **(B)** the acupoints selected in the SA group. Created with BioRender.com.

The acupoint positions in both groups are depicted in Figure 1.

### 2.4. Randomization and blinding

Randomization was conducted with a block size of six. Random numbers generated by the SAS statistical analysis system were sealed in opaque envelopes. A research assistant who was not involved in the intervention or assessment of the trial was in charge of the randomization. Owing to the characteristics of acupuncture, acupuncturists were not blinded to the assignments. The non-effective acupoints acupuncture design can however guarantee a good blinding effect for the participants. Assessors and statisticians involved in data collection and analysis were blinded to the assignments.

### 2.5. Assessment scale

The PSQI consists of 19 self-assessed and five other-assessed items. It is used to assess the sleep quality of the subjects over the last month. The higher the PSQI score, the worse the sleep quality (Buysse et al., 1989). The Hyperarousal Scale (HAS) consists of 26 arousal-related items and is used to assess cortical hyperarousal states, with higher scores indicating higher levels of arousal (Pavlova et al., 2001). The PSQI and HAS scales were assessed before and after treatment.

### 2.6. Actigraphy

Participants were required to wear an actigraphy (MTI Health Services Company, Pensacola, FL, United States) on their non-dominant wrist to obtain objective data on sleep such as total sleep time (TST), the time of wake after sleep onset (WASO), and sleep efficiency (EFFICIENCY). The device was worn for a week before and after treatment.

### 2.7. Imaging acquisition

MRI scans were performed on a 3.0-Tesla MRI scanner (MAGNETOM, Trio, Siemens, Germany) at the Beijing Hospital of Traditional Chinese Medicine. High-resolution whole-brain structural images were recorded using a T1-weighted isotropic multi-echo magnetization-prepared rapid acquisition gradient-echo (MPRAGE) pulse sequence. Resting-state functional images were obtained by blood oxygen level dependent (BOLD) echo-planar imaging (EPI) sequences. To correct for the effects of magnetic field inhomogeneity, a field map sequence was scanned before EPI (Jezzard and Balaban, 1995). Further details are provided in Supplementary Table S1. During scanning, subjects were required to stay awake and avoid thinking about anything.

### 2.8. Image processing and analysis of fMRI data

Image pre-processing and FC analysis were conducted by Data Processing & Analysis of Brain Imaging (DPABI, http://rfmri.org/dpabi) (Yan et al., 2016), a toolbox in MATLAB 2021b (The MathWorks, Inc.). After removing the first 10 image points of each participant to eliminate the effect of uneven magnetic fields at the beginning or participant discomfort with the scan, the main steps of pre-processing included: slice-timing, realignment, normalization to the Montreal Neurological Institute (MNI) coordinate space with 3 × 3 × 3 mm^3^, smooth with a 6 × 6 × 6 mm^3^ full-width at half maximum Gaussian kernel, linear detrending; band-pass filtering (0.01–0.1 Hz), and nuisance signals were regressed out, including the Friston 24 head motion parameters (Friston et al., 1996), global signal, white matter signal, and cerebrospinal fluid signal.

After pre-processing, the LC, derived from automated anatomical labeling 3 (AAL3) (Rolls et al., 2020) was selected as the region of interest (ROI) for FC analysis. First-level correlation maps were produced by extracting the BOLD time course from the ROI and computing Pearson’s correlation coefficients between that time course and the time courses of all other voxels in the brain. Then correlation coefficients were Fisher transformed into z scores to increase normality.

Whole-brain second-level group analysis was applied using paired t-tests for within-group comparisons before and after treatment and sample t-tests for between-group comparisons. Age, gender, and head movement parameters were included as covariates of non-interest. A Gaussian random field (GRF) correction at a threshold of *p*-voxel < 0.001 and *p*-cluster < 0.05 was applied. Brain regions with differences were visualized with the BrainNet Viewer^1^ (Xia et al., 2013).

### 2.9. Statistical analysis

The trial data were analyzed according to the per-protocol principle, and patients who completed the two fMRI scans were included in the data analysis. Normality was assessed by the Shapiro–Wilk test. Categorical variables are represented as counts and percentages, and continuous variables as means with standard deviations (SD) or medians with interquartile ranges (IQR). Differences in continuous variables were compared with the use of a Student’s *t*-test or Mann–Whitney U test. Categorical outcomes were compared with the use of a chi-square test. A two-sided *p* value of less than 0.05 was considered to indicate statistical significance. Pearson’s correlation analysis was used to investigate correlations between clinical outcome changes (post- minus pre-) and the ΔFC z-score (post- minus pre-). The Bonferroni method was used for multiple comparison correction, *p* < 0.01 (0.05/5) was considered to be statistically significant. Analyses were performed with the use of SPSS software, version26.0 (International Business Machines Corporation).

## 3. Results

Detailed baseline demographic and clinical characteristics are shown in Table 1. No significant difference between groups was observed (all *p* > 0.05).

**Table 1:** Demographic and clinical characteristics of ID patients.

Demographic characteristics		RA (*N* = 26)	SA (*N* = 24)	*p*-value
Age^a^		31.50 (18)	40.00 (20)	0.240
Sex^b^	Male	8 (30.77%)	10 (41.67%)	0.423
	Female	18 (69.23%)	14(58.33%)	
Educational levels^b^	Below bachelor’s degree	5 (20.7%)	10 (40.0%)	0.084
	Bachelor’s degree or above	21 (79.3%)	14 (60.0%)	
PSQI		12.46 ± 2.27	12.50 ± 2.36	0.953
HAS		43.88 ± 7.76	42.88 ± 7.62	0.245
Actigraphy	TST (min)	390.23 ± 48.27	387.00 ± 66.20	0.854
	WASO (min)^a^	83.00 (55)	65.67 (53)	0.697
	EFFICIENCY (%)^a^	78.63 (10)	83.98 (10)	0.119

Unless otherwise indicated, data are represented as mean ± SD, and the *p*-values were obtained by using a *t*-test. PSQI, Pittsburgh Sleep Quality Index; HAS, Hyperarousal Scale; TST, total sleep time; WASO, wake after sleep onset.

a Data are represented as median and IQR, and the value of ps were obtained by using a Mann–Whitney *U* test.

b Data are represented as counts and percentages, and the value of ps were obtained by using a Chi-square test.

### 3.1. Clinical data

After 4 weeks of acupuncture, the ΔPSQI score was −4.73 ± 3.47 in the RA group and − 1.25 ± 2.56 in the SA group, with a between-group difference of −3.48 ± 0.86 (*p* < 0.001); the median of the ΔHAS score was −10.00 in the RA group and − 1.00 in the SA group, with a between-group difference (*Z* = 0.381, *p* < 0.001). Similarly, the sleep efficiency in the RA group after treatment was significantly improved compared with the baseline period and the SA group (all *p* < 0.05). The specific data is shown in Tables 2, 3.

**Table 2:** Results of statistical analysis of clinical outcome after treatment (intra- and inter-group).

Clinical outcome	RA	SA	*p*_1_	*p*_2_	*p*_3_
PSQI	7.73 ± 3.24	11.25 ± 2.25	<0.001^***^	0.025^*^	<0.001^***^
HAS	33.42 ± 10.21	41.38 ± 9.23	<0.001^***^	0.167	0.006^**^
TST (min)	404.70 ± 55.96	380.14 ± 52.76	0.328	0.476	0.148
WASO (min)^a^	50 (49)	58.92 (48)	0.085	0.445	0.601
EFFICIENCY (%)	87.70 ± 4.10	83.67 ± 7.04	<0.001^***^	0.185	0.032^*^

Unless otherwise indicated, data are represented as mean ± SD, and the *p*-values were obtained by using a *t*-test. PSQI, Pittsburgh Sleep Quality Index; HAS, Hyperarousal Scale; TST, total sleep time; WASO, wake after sleep onset. *p*1: compared with baseline in RA group; *p*2: compared with baseline in SA group; *p*3: compared between RA and SA groups. *: *p* < 0.05; **: *p* < 0.01; ***: *p* < 0.001.

a Data are represented as median and IQR, and the value of ps were obtained by using a Mann–Whitney *U* test.

**Table 3:** Results of statistical analysis of clinical outcome changes (post- minus pre-) between groups.

Clinical outcome changes	RA	SA	*p*
ΔPSQI	−4.73 ± 3.47	−1.25 ± 2.56	<0.001^***^
ΔHAS^a^	−10.00 (9)	−1.00 (7)	<0.001^***^
ΔTST (min)	14.47 ± 69.40	4.96 ± 48.67	0.611
ΔWASO (min)^a^	−14.50 (46.83)	−8.00 (27.46)	0.436
ΔEFFICIENCY (%)^a^	7.53 (9.15)	2.73 (6.60)	0.006^**^

Unless otherwise indicated, data are represented as mean ± SD, and the value of ps were obtained by using a *t*-test. PSQI, Pittsburgh Sleep Quality Index; HAS, Hyperarousal Scale; TST, total sleep time; WASO, wake after sleep onset. *: *p* < 0.05; **: *p* < 0.01; ***: *p* < 0.001.

a Data are represented as median and IQR, and the *p*-values were obtained by using a Mann–Whitney *U* test.

### 3.2. fMRI data

No brain regions were found different between groups at baseline at the threshold we set.

**Figure 2:**
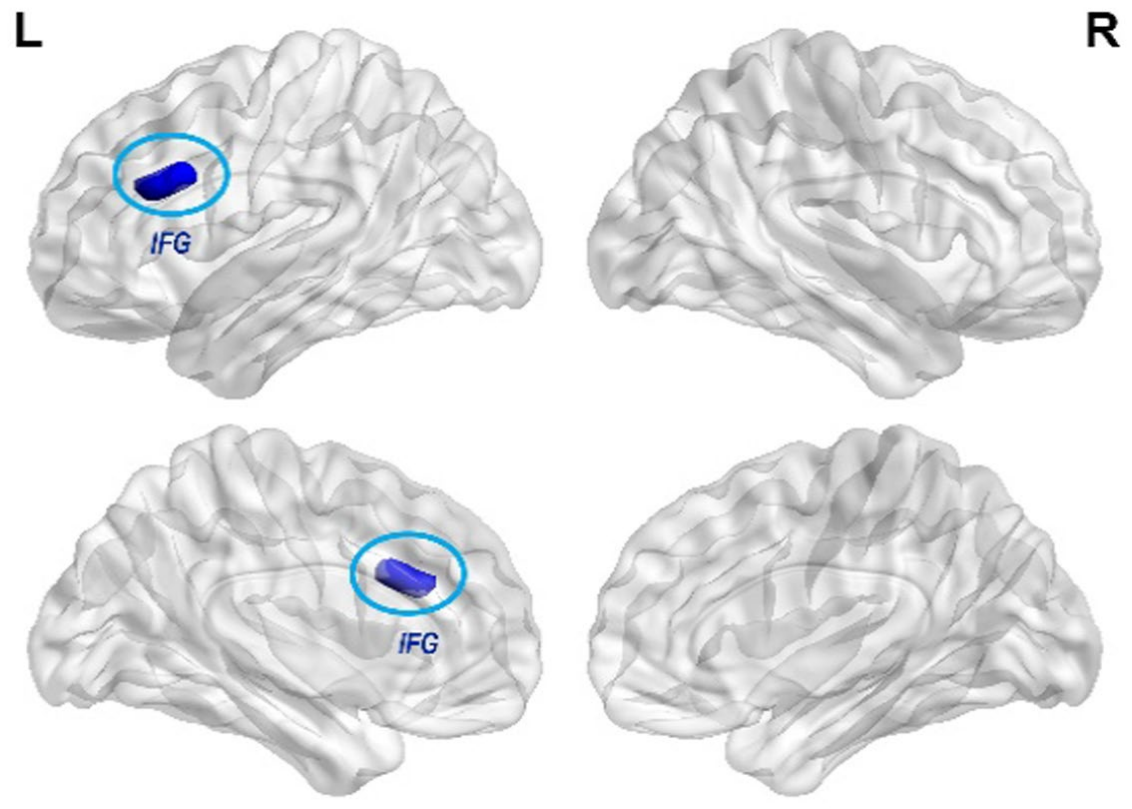
Changes of LC FC in the RA group compared with baseline. The blue color indicates the LC -left IFG FC is decreased in RA after treatment. (GRF correction, *p*-voxel < 0.001, *p*-cluster < 0.05, cluster size > 21).

Compared with baseline, the LC FC with the left inferior frontal gyrus (IFG) was decreased in RA after treatment (Table 4; Figure 2), and no FC changes in any brain region in the SA groups were found at the threshold we set.

**Table 4:** Brain regions showing decreased FC in RA after treatment compared with baseline.

Clusters	MNI coordinates			Voxels	AAL Brain Region	*P*eak*t* value
	X	Y	Z			
Left inferior frontal gyrus	−45	18	30	34	Frontal_Inf_Tri_L	−6.21

GRF correction, P-voxel < 0.001, P-cluster < 0.05, cluster size > 21. AAL, Anatomical Automatic Labeling.

**Figure 3:**
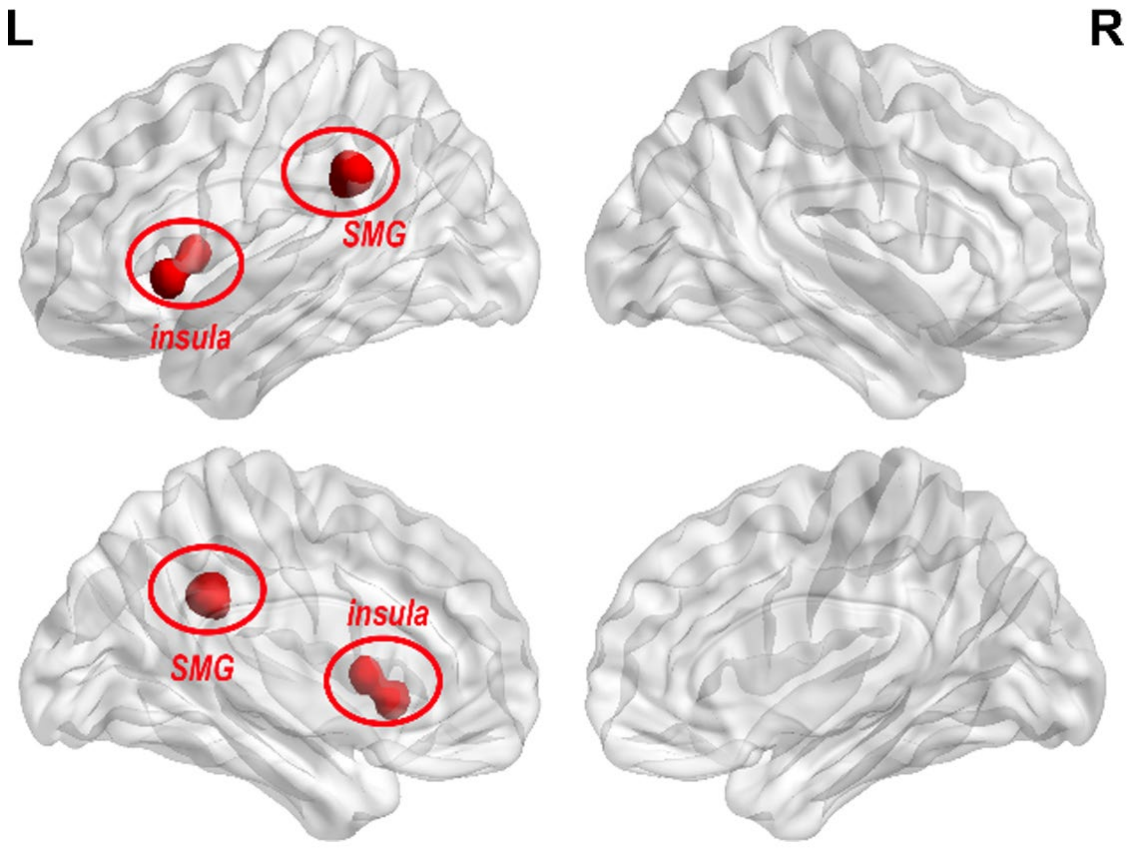
Changes of LC FC in the RA group compared with the SA group after treatment. The red color in the figure indicates the brain areas with LC FC increases in the RA group compared with the SA group (GRF correction, *p*-voxel < 0.001, *p*-cluster < 0.05, cluster size > 26).

In the post-treatment between-group comparison, the LC FC of the left insula and supramarginal gyrus (SMG) was higher than that in the SA group (Table 5; Figure 3). Intergroup comparisons revealed that ΔFC values of LC-SMG were statistically different in brain regions (*p* < 0.05) (Table 6).

**Table 5:** Brain regions showing stronger FC in RA compared with SA.

Clusters	MNI coordinates			Voxels	AAL Brain Region	Peak *t* value
	X	Y	Z			
Left insular	−39	18	−3	32	Insula_L	4.772
Left supramarginal gyrus	−60	−42	33	30	SupraMarginal_L	4.857

GRF correction, P-voxel < 0.001, P-cluster < 0.05, cluster size > 26. AAL, Anatomical Automatic Labeling.

**Table 6:** Intergroup comparisons of ΔFC values in brain regions with differences.

	RA	SA	*t* value	*p*-value
ΔFC of insula	−0.01 ± 0.20	−0.10 ± 0.15	1.87	0.067
ΔFC of SMG	0.01 ± 0.21	−0.11 ± 0.21	2.15	0.037^*^

SMG, supramarginal gyrus. *: *p* < 0.05.

**Figure 4:**
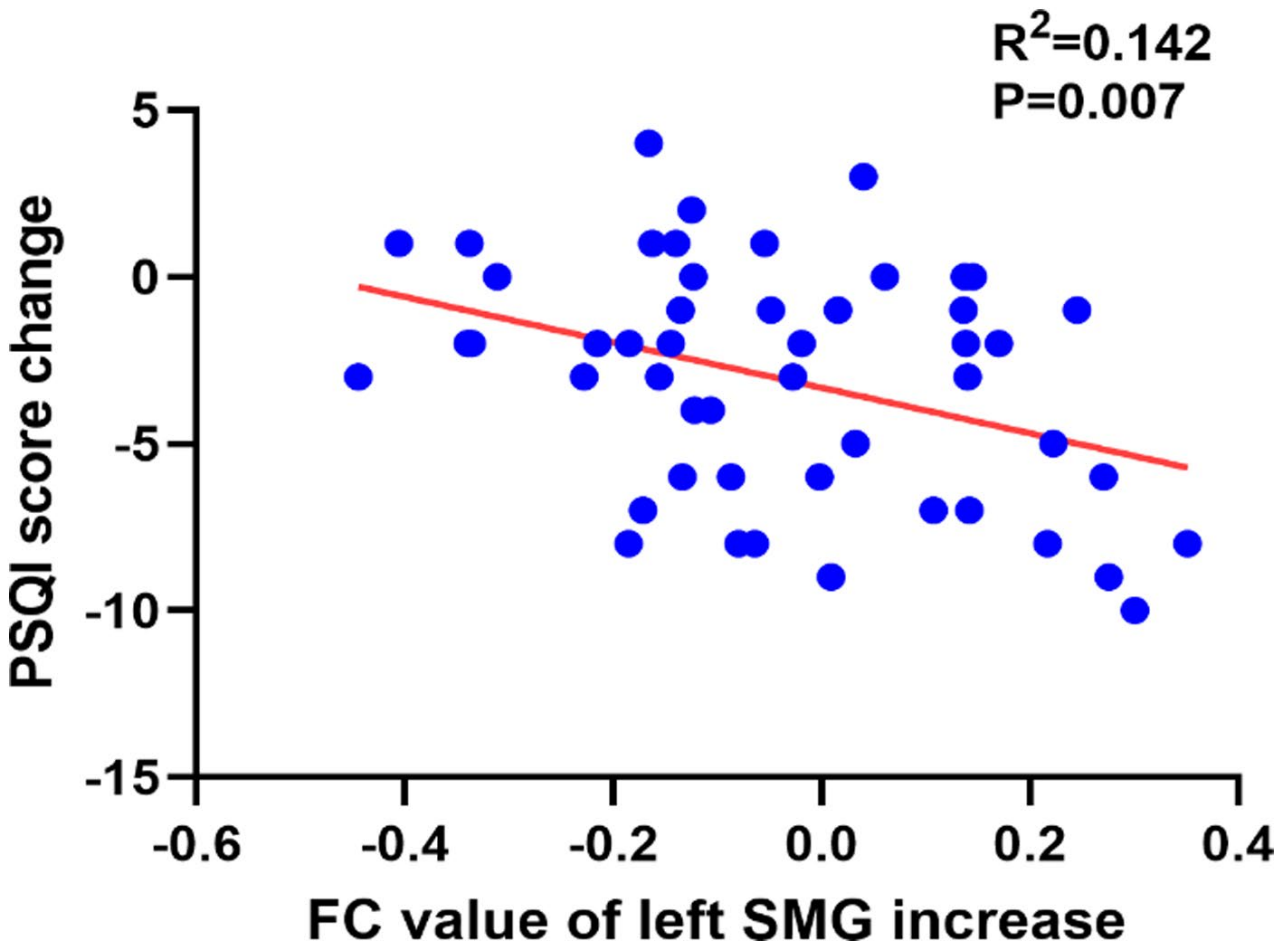
Analysis of the correlation between the FC changes and PSQI improvement in all subjects.

The results of the correlation analysis results showed that ΔPSQI was negatively associated with the ΔFC value of the LC- left SMG in both RA (r = 0.432, *p* = 0.028) and all subjects (*r* = −0.377, *p* = 0.007), and was negatively associated with the ΔFC value of the LC-left insula (*r* = −0.279, *p* = 0.049). After Bonferroni correction, only the correlation between ΔPSQI and ΔFC value of the LC-left SMG in all subjects was statistically significant (*p* < 0.01) (Figure 4). No correlation was found between ΔFC and clinical outcome changes (all *p* > 0.01) (Supplementary Table S2).

## 4. Discussion

This study aims to explore the effect of acupuncture on the FC of LC in ID patients. The clinical data suggested that acupuncture could modulate the state of arousal and improve sleep quality in insomniacs. Sleep quality was improved in both groups, but more significantly in the RA group. The doctor-patient interaction during acupuncture treatment may relieve the patients’ negative emotions in both groups (Liu, 2007). The fMRI analysis revealed that the FC between the LC and the IFG, insular, and SMG were modulated following acupuncture treatment.

The IFG is considered a key brain region for sleep. Individuals with lower gray matter density in the left IFG are more likely to develop insomnia and wake early (Stoffers et al., 2012). Studies have shown that the activity of the IFG decreases during sleep while increases during sleep deprivation (Vartanian et al., 2014). Li et al. discovered that the amplitude of the low-frequency fluctuation value of the left IFG was reduced in insomnia patients and negatively correlated with the duration of insomnia (Li et al., 2016). Yan et al. discovered that the degree centrality value of the left IFG decreased proportionally to the PSQI score in insomniacs (Yan et al., 2018). The IFG is a fundamental component of the cognitive control network (CCN), which is involved in executive function and cognitive control (MacDonald et al., 2000). In a previous study, we discovered that patients with insomnia had higher LC FC in the middle frontal gyrus than healthy people (Chen et al., 2022). Both the middle frontal gyrus and IFG belong to CNN, demonstrating the importance of CNN in sleep regulation. Abnormal CCN function was found in ID patients, which may account for the heightened sensitivity to external stimulation (Nofzinger, 2004). Our study found that the FC between the LC and IFG was downregulated after acupuncture, which is consistent with the previous findings; thus, acupuncture may inhibit the overactivation of the IFG.

The insula is part of the salience network (SN) and is crucial for decision-making, cognition, emotion regulation, and attention control (Uddin, 2015). Patients with ID exhibited abnormal insular activity during both task execution and resting states. ID patients showed decreased FC between the insula, amygdala, striatum, and thalamus compared to normal sleepers (Martin et al., 2015; Lu et al., 2017). The co-activation of the insula with the SN is increased when ID patients fall asleep (Chen et al., 2014). Prior studies found that the FC value between the bilateral insula in ID patients decreased (Huang et al., 2017; Liu et al., 2018). The anatomical connection between the LC and insula is related to the processing of unexpected events (Dayan and Yu, 2006; Eschenko et al., 2012), and the changes in the FC of the LC-insula may be associated with the modulation of perceptual awareness skills and salience event processing (Liu J. et al., 2021). Our study found the consistent results, real acupuncture could increase the FC of the LC-left insular. But we were unable to establish a significant correlation between this finding and clinical assessments, which may be related to the sample size. Based on the above studies, we speculated that acupuncture may be beneficial for interoceptive and emotional processing in ID by modulating the abnormal functional of the insula.

The ΔFC value of SMG showed differences between groups. And ΔPSQI was negatively associated with the ΔFC value of LC-left SMG. The SMG belongs to the inferior parietal lobule, which is part of the DMN (Husbani et al., 2021) and plays an important role in the adjustment of consciousness (Marques et al., 2018). Kay et al. discovered an inverse correlation between the rate of glucose metabolism in SMG and sleep efficiency (Kay et al., 2016). A voxel-based morphometry study showed that the white matter volume of SMG was negatively associated with the PSQI score (Bai et al., 2022), and the higher volume of SMG was correlated with the longer sleep duration (Cheng et al., 2021). Studies found that the increasement in parietal lobe activity could compensate for the decreased cognition caused by insomnia (Chee and Choo, 2004; Li Y. et al., 2022). Based on these findings, we believe that acupuncture may relieve cognitive decline caused by insomnia by modulating LC-SMG FC.

An earlier study found decreased LC-IFG FC and elevated LC-SMG FC in ID patients (Gong et al., 2021), which appears inconsistent with our findings. This difference may be related to the compensatory mechanism described above and, on the other hand, to the variation in depression and anxiety in the included population. Anxiety and depression were found to affect the FC of IFG and brain regions in DMN (Fang et al., 2016; Yan et al., 2019; Yu et al., 2021). Therefore, it is difficult to determine whether insomnia or anxiety and depression had a greater impact on the FC of LC-IFG and LC-SMG in that study. The FC of LC is also influenced by age, differences in demographic characteristics can also contribute to inconsistent results (Zhang et al., 2016; Song et al., 2021). In addition, the difference in the method of LC localization may also play roles. There are relatively few studies on the FC of LC in insomnia patients, and the results lack uniformity (Gong et al., 2021; Li C. et al., 2022). To obtain more accurate results, a big-scale study focused on ID patients without emotional problems is needed. As for the brain regions that showed differences in the between-group comparisons but did not appear in the within-group comparisons (insula and SMG), it may be related to the non-specific effect of the sham acupuncture, which needs to be further explored. Furthermore, we discovered that the brain regions with FC differences are in the left hemisphere, which is intriguing. We suspect this is because the insomnia patients we recruited were all right-handed. Activation in the left hemisphere of the brain was found to be more dominant than the right in right-handed people (Gut et al., 2007). We will include left-handed patients in our future study to test our hypothesis.

Acupuncture has been found to regulate heart rate variability, indicating that acupuncture can inhibit the sympathetic nerve and activate the vagus nerve (Wang et al., 2002; da Silva and Dorsher, 2014). Acupuncture’s regulation mechanism on the LC FC may be related to afferent vagus nerve stimulation. The nucleus of the solitary tract (NTS) is primarily innervated by vagal afferents, and the NTS projects to the locus coeruleus. The peripheral vagus nerve can be stimulated to further regulate the functional activities of the LC. Future research should be combined with heart rate variability and other indicators to evaluate vagal nerve activity in order to validate this hypothesis.

There are some limitations in our research. First of all, the LC is the main source of NE, but this study did not involve the measurement of NE, and future studies should be combined with relevant examinations. Second, actigraphy relies on exercise to identify the awake period, although it is relatively objective but not accurate enough, polysomnography should be adopted. Thirdly, the LC has only 45,000–50,000 cells, making its precise location difficult. Even though the LC region has been added to AAL3, a more precise map of the Chinese brain is required. Additionally, the sample size was relatively small in this study. Additional studies should be conducted with larger sample sizes to confirm our findings. Lastly, there was no follow-up in this study, in the future, a long-term observation is required.

## 5. Conclusion

To our knowledge, this is the first study to investigate the mechanism of acupuncture treatment of insomnia utilizing the FC of LC. We found that the behavioural results of the insomniacs improved after acupuncture, and the imaging results showed that the FC between the LC and IFG, insular and SMG were modulated. The LC, which connects the SN, CCN, and DMN, may be an important target for acupuncture to improve insomnia.

## Data availability statement

The raw data supporting the conclusions of this article will be made available by the authors, without undue reservation.

## Ethics statement

The studies involving human participants were reviewed and approved by Medical Ethical Committee of the Beijing Hospital of Traditional Chinese Medicine. The patients/participants provided their written informed consent to participate in this study.

## Author contributions

ZC participated in the conception and design of this study, data collection, organization, and drafting of the paper, and revised the content of the paper. TJ and XY participated in the design, organization, and analysis of this study. ZT participated in the fMRI experimental design. JG and BL participated in the design and implementation of the experiment, participated in the analysis and interpretation of the data, provided guidance on the experiment, provided administrative, technical, and material support, and made key revisions to the academic content of the paper. All authors contributed to the article and approved the submitted version.

## Funding

This study was funded by the National Natural Science Foundation of China (81774391), Natural Science Foundation of Beijing Municipality (7212170) and the Beijing Key Laboratory of Acupuncture Neuromodulation, Beijing, China (BZ0437).

## Conflict of interest

The authors declare that the research was conducted in the absence of any commercial or financial relationships that could be construed as a potential conflict of interest.

## Publisher’s note

All claims expressed in this article are solely those of the authors and do not necessarily represent those of their affiliated organizations, or those of the publisher, the editors and the reviewers. Any product that may be evaluated in this article, or claim that may be made by its manufacturer, is not guaranteed or endorsed by the publisher.

## Footnotes

^1^ http://www.nitrc.org/projects/bnv/
