# Supplementary material for: The increased functional connectivity between the locus coeruleus and supramarginal gyrus in insomnia disorder with acupuncture modulation
Source: Front Neurosci. 2023 Apr 20;17:1131916. doi: 10.3389/fnins.2023.1131916 (PMC10157050; doi:10.3389/fnins.2023.1131916)
Supplement: Supplementary file 1 [file Data_Sheet_1.PDF]

Supplement1.

Imaging acquisition parameters

Sequence	MPRAGE	EPI	field map
**Field of View (FOV)/ mm^2**	256 × 256	224 × 224	224 × 224
**Matrix**	256 × 256	64×64	64×64
**Slice Thickness/mm**	1	3.5	3.5
**Gap/mm**	0	0.7	0.6
**Resolution/mm^3**	1 × 1 × 1	3.5 ×3.5 × 3.5	3.5 ×3.5 × 3.5
**Slice Number**	188	32	32
**Repetition Time (TR)/ms**	2000	2000	400
**Echo Time (TE)/ms**	3.51	30	5.19
**Flip Angle/degree**	7	90	60
**Phase Direction**	Anterior to Posterior	Anterior to Posterior	Anterior to Posterior
**volume**	1	240	1
**Acquisition Time (TA)**	4m50s	8m6s	54s
