# Supplementary material for: The increased functional connectivity between the locus coeruleus and supramarginal gyrus in insomnia disorder with acupuncture modulation
Source: Front Neurosci. 2023 Apr 20;17:1131916. doi: 10.3389/fnins.2023.1131916 (PMC10157050; doi:10.3389/fnins.2023.1131916)
Supplement: Supplementary file 2 [file Data_Sheet_2.PDF]

Supplement 2

Table1.Correlation analysis between clinical outcome changes and ΔFC in the RA group

	ΔFC of IFG		ΔFC of insula		ΔFC of SMG	
	r	p	r	p	r	p
**ΔPSQI**	0.039	0.852	-0.321	0.110	-0.432	0.028*
**ΔHAS**	-0.027	0.897	-0.062	0.762	0.146	0.478
**ΔTST**	-0.064	0.772	0.072	0.744	0.036	0.871
**ΔWASO**	-0.165	0.451	0.055	0.803	0.345	0.107
**ΔEFFICIENCY**	-0.056	0.800	0.118	0.590	0.052	0.815

*: p<0.05

Table2.Correlation analysis between clinical outcome changes and ΔFC in the SA group

	ΔFC of IFG		ΔFC of insula		ΔFC of SMG	
	r	p	r	p	r	p
**ΔPSQI**	-0.172	0.421	-0.004	0.984	-0.088	0.683
**ΔHAS**	-0.138	0.519	0.195	0.360	0.139	0.517
**ΔTST**	-0.032	0.895	-0.097	0.685	-0.004	0.986
**ΔWASO**	-0.030	0.900	-0.232	0.325	-0.248	0.293
**ΔEFFICIENCY**	0.032	0.892	0.240	0.307	0.237	0.315

Table3.Correlation analysis between clinical outcome changes and ΔFC in all subjects

	ΔFC of IFG		ΔFC of insula		ΔFC of SMG	
	r	p	r	p	r	p
**ΔPSQI**	0.146	0.313	-0.279	0.049*	-0.377	0.007**
**ΔHAS**	0.150	0.299	-0.100	0.489	0.003	0.981
**ΔTST**	-0.070	0.658	0.029	0.854	0.032	0.838
**ΔWASO**	-0.037	0.813	-0.085	0.590	0.089	0.572
**ΔEFFICIENCY**	-0.040	0.797	0.201	0.195	0.169	0.278

*: p<0.05, **: p<0.01
